# Opportunities and challenges for advance care planning in strongly religious family-centric societies: a Focus group study of Indonesian cancer-care professionals

**DOI:** 10.1186/s12904-022-01002-6

**Published:** 2022-06-22

**Authors:** Diah Martina, Christina Yeni Kustanti, Rahajeng Dewantari, Noorwati Sutandyo, Rudi Putranto, Hamzah Shatri, Christantie Effendy, Agnes van der Heide, Judith A. C. Rietjens, Carin van der Rijt

**Affiliations:** 1 Department of Medical Oncology, Erasmus MC Cancer Institute, University Medical Center Rotterdam, Dr. Molewaterplein 40, 3015 GD Rotterdam, the Netherlands; 2 Department of Public Health, Erasmus MC, University Medical Centre Rotterdam, Rotterdam, the Netherlands; 3 Division of Psychosomatic and Palliative Medicine, Department of Internal Medicine, Universitas Indonesia, Jakarta, Indonesia; 4 Cipto Mangunkusumo National General Hospital, Jakarta, Indonesia; 5 Bethesda Yakkum Institute for Health Sciences, Yogyakarta, Indonesia; 6 Department of Neuro-Psychiatry, Dharmais National Cancer Center, Jakarta, Indonesia; 7 Department of Hematology and Medical Oncology, Dharmais National Cancer Center, Jakarta, Indonesia; 8 School of Nursing, Faculty of Medicine, Public Health and Nursing, Universitas Gadjah Mada, Yogyakarta, Indonesia

**Keywords:** Advance care planning, Oncology, Asia, Culture, Spirituality, Health personnel

## Abstract

**Background:**

Most studies on advance care planning in Asia originate in high-income Asian countries. Indonesia is a middle-income Asian country characterized by its religious devoutness and strong family ties. This study aims to explore the perspectives and experiences of Indonesian healthcare professionals on advance care planning for cancer patients.

**Methods:**

Focus-group discussions were conducted in July and August 2019 and were analysed using thematic content analysis enhanced by dual coding and exploration of divergent views. Purposive sampling of physicians and nurses actively engaged in cancer care in a national cancer centre and a national general hospital.

**Results:**

We included 16 physicians and 16 nurses. These participants were open to the idea of advance care planning. We further identified four aspects of this planning that the participants considered to be important: 1) the family’s role in medical decision-making; 2) sensitivity to communication norms; 3) patients’ and families’ religious beliefs regarding the control and sanctity of life; and 4) the availability of a support system for advance care planning (healthcare professionals’ education and training, public education, resource allocation, and formal regulation). Participants believed that, although family hierarchical structure and certain religious beliefs may complicate patients’ engagement in advance care planning, a considerate approach to involving family and patients’ religious perspectives in advance care planning may actually facilitate their engagement in it.

**Conclusion:**

Indonesian healthcare professionals believed that, for culturally congruent advance care planning in Indonesia, it was essential to respect the cultural aspects of collectivism, communication norms, and patients’ religious beliefs.

**Supplementary Information:**

The online version contains supplementary material available at 10.1186/s12904-022-01002-6.

## Introduction

Advance care planning is a process in which patients reflect upon the meanings and consequences of serious illness. It enables them to identify their values, and define their goals and preferences for future care, and discuss them with their family and healthcare professionals [1]. A recent review of studies from Western countries showed that it improves patients’ and surrogates’ satisfaction with communication, and reduces surrogates’ and clinicians’ distress [2].

Interest and research in advance care planning have been growing not only in Western countries [3], but also in Eastern ones [4–6]. Our review on advance care planning in southern, south-eastern, and eastern Asian countries showed that even though Asian healthcare professionals acknowledge its importance, and are willing to engage in it, they find it very challenging to do so [6]. However, while most research on advance care planning in Asia has been conducted in high-income countries [3, 7], few studies have examined it in low and middle-income Asian countries, including Indonesia [7].

Indonesia is the fourth most populous country in the world whose incidence of cancer has increased 29% over the last five years since 2013 [8]. Seventy percent of these patients are at an advanced stage, where advance care planning is especially important to enabling their autonomy at the end of life [8]. However, the uptake of advance care planning may be influenced by Indonesia’s culture of collectivism, in which family plays a major role in medical decision-making [9, 10]. In addition to this, it may be further influenced by most Indonesian people’s religious devotion [10–14].

A recent survey among the general population showed that 75% of Indonesians were willing to engage in end-of-life care conversations, and 60% of them expected healthcare professionals to initiate it [15]. However, a study on the actual use of advance care planning and the potential facilitators and challenges faced by healthcare professionals has not been performed in Indonesia. This study therefore aimed to better understand Indonesian healthcare professionals’ perspectives on and experiences with advance care planning in oncology care by conducting exploratory focus-group discussions.

## Methods

### Study design

Focus-group discussions were conducted to enable active interaction between participants and to stimulate clarification of views and sharing of various perspectives on and experiences with advance care planning that might otherwise be less evident in the context of individual interviews [16]. The interpretative phenomenological analysis approach was used to study how phenomena appear to the subject and how his or her experience is established [17]. Reporting was guided by the Consolidated Criteria for Reporting Qualitative Research (COREQ) [18].

### Study setting

The study was conducted in the only national cancer centre in Indonesia and in a top-referral academic general hospital, both situated in Jakarta, the capital city of Indonesia.

### Sampling and recruitment

Physicians and nurses who were actively engaged in the treatment and care of patients with cancer were invited to participate. To capture the diversity of clinical specialties, age, and gender, participants were then purposively sampled. Participants at Dharmais National Cancer Centre were selected and invited by RD, and participants at Cipto Mangunkusumo National General Hospital by DM. Specific inclusion criteria included: (1) experience for at least five years with the provision of care to patients with cancer, and (2) the provision of informed consent.

### Focus-group discussions and data collection

All focus-group discussions were moderated by DM (Indonesian female researcher and physician specializing in internal medicine and palliative care, trained in performing qualitative studies), who also encouraged group members to exchange opinions. The discussions were observed and recorded by RD (Indonesian female physician specialized in psychiatry and palliative care, experienced in qualitative studies), who also made additional notes based on her observations.

Before starting these discussions, we developed a topic guide based on our systematic reviews of advance care planning in Asia [5, 6] and on consultation of various experts on palliative and cancer care, with backgrounds in medical oncology, palliative care, research, and psychology. The focus-group topic guide (Appendix 1) addressed: (1) an introduction to the study; (2) participants’ prior knowledge of advance care planning; (3) participants’ perspectives on advance care planning; (4) whether and how advance care planning was practiced at participants’ current workplace, and their ideas about it; and (5) barriers and facilitators for advance care planning. Due to the lack of an Indonesian term for or concept of advance care planning, the concept used in this study was consistent with the international consensus definition of the European Association for Palliative Care: “a process that enables individuals with decisional capacity to identify their values, to reflect upon the meanings and consequences of serious illness scenarios, to define goals and preferences for future medical treatment and care, and to discuss these with family and healthcare professionals, and to record and review these preferences if appropriate.” [1].

### Data processing and analysis

All discussions were audio-recorded and transcribed verbatim in Indonesian, the official language at the study sites, by DM and RD. DM and CYK (Indonesian female nurse and researcher, trained and experienced in qualitative studies, and fluent in English) then analysed the data following the inductive thematic analysis approach [19]. First, DM and CYK familiarized themselves with the data by reading the transcripts several times before identifying ideas. Second, DM and CYK independently generated initial codes by allocating codes to these ideas. Third, DM and CYK independently grouped the codes under broader themes. To achieve consensus, the generation of codes and themes was followed by discussions between DM and CYK. To enhance the validity and confirmability of the findings, we performed investigator triangulation (DM, CYK, CE, JR, AH, and CR), by translating codes and themes into English to facilitate the discussions with the non-Indonesian-speaking co-authors (JR, AH and CR). Prior to these discussions, DM and CYK selected two transcripts based on the richness, had them translated into English by a professional translator, and shared them with JR, AH, and CR. In the fourth phase, meetings were held between DM, CYK, CE, JR, AH, and CR to review the themes and ensure each theme had a specific identity. All of these processes were iterative and reflective, developing over time and involving a constant moving back and forward between phases. To assist in data management, N-Vivo qualitative data analysis software (version 12) was used.

## Results

### Participants’ demographics

We included 16 nurses and 16 physicians and held five focus-group discussions between July and August 2019. We conducted two focus-group discussions in Dharmais (one with seven physicians and one with eight nurses); and three in Cipto Mangunkusumo: (one with four physicians, one with five physicians and one with eight nurses). Each discussion lasted approximately 90 min. We terminated the data collection after discovering no additional data that would add further insights to the findings. The participants’ characteristics are summarized in Table 1.

**Table 1:** Characteristics of the participants

	**Physicians (*N*** **= 16)**	**Nurses (*N*** **= 16)**
**Sex**		
Male	10	5
Female	6	11
**Age (years)**		
< 40	6	14
40–60	9	1
> 60	1	1
**Specialty**		
Medical oncology	3	
Surgical oncology	2	
Neuro-oncology	1	
Pulmonology	1	
Geriatrics	2	2
Anaesthesiology (intensive care)	2	2
Palliative care	2	1
Head and neck oncology	1	
Uro-oncology	1	
Hepato-gastroenterology	1	
Oncology		11

### Thematic findings

**Fig. 1:**
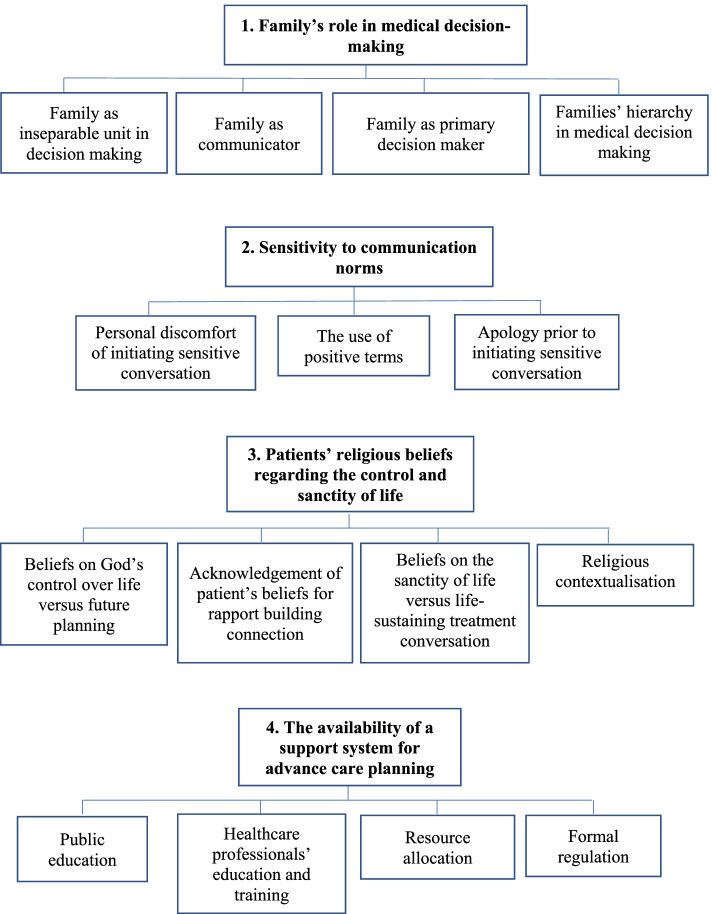
Coding Tree

Four main themes were identified as key features of healthcare professionals’ perspectives on and experiences with advance care planning (Fig. 1): 1) family’s role in medical decision-making; 2) sensitivity to communication norms; 3) patients’ religious beliefs regarding the control and sanctity of life; and 4) the availability of a support system for advance care planning.

#### Theme 1: Family’s role in medical decision-making

In Indonesia, many elderly patients live together with their children, and participants often reported that patients greatly appreciated the input from their family members.

*“Patients are often not capable of making decisions [by themselves]. They’ll say, ‘let me ask my family first, Doc.’” (P04: male, head-and-neck oncologist)*

Due to the importance of families in medical decision-making, participants believed that gaining their support in advance care planning is essential. Families may also serve as patients’ proxies for collecting meaningful information.

*“Some patients may have communicated their wishes to the family… as already mentioned, the role of family in Indonesia is very dominant, and they may be the ones we approach before the patient… that is the reality. Some patients have already shared their wishes in detail with their children. By involving the family, much information can be obtained, but not all of it will be verified with the patient. I usually only clarify the major things.” (P09: female, geriatrician)*

Nevertheless, participants stated that patients were particularly deprived of opportunities for meaningfully engaging in advance care planning if their family took the leading role in decision-making.

*“What needs to be underlined in our culture in Indonesia is that patients often cannot determine their fate, because their family will decide [for them].” (N12: male, oncology nurse)*

Participants described that disclosing bad news was a necessary yet difficult precondition for advance care planning. Families’ reluctance to inform patients about their poor prognosis contributed to missed opportunities for the timely engagement of patients in advance care planning.

Certain families’ structures and dynamics, in which hierarchy played an important role, were reported to potentially complicate advance care planning, in ways that sometimes prevented patients’ wishes from being acted upon.

*“The family member who takes care of the patient closely can usually understand his suffering and respect his wishes. But once another family member with a higher position or who is more respected [by other members of the family] comes, he/she may cancel everything [that has been agreed upon].” (N05: male, oncology nurse)*

#### Theme 2: Sensitivity to communication norms

Due to the sensitive nature of advance care planning conversations, many participants felt uncomfortable about initiating them. They also reported that some patients may not appreciate them.

*“I don’t have the heart to talk about it [advance care planning]. Our patients are also very rarely willing to engage in such a conversation.” (P03: female, medical oncologist)*

Most participants felt the need to know how to approach advance care planning in a way that would be acceptable to patients and their families. The use of positive terms was reported as being more appreciated by them.

*“We would not use the term ‘refusal of resuscitation’ rather than ‘allow natural death’ when asking them to sign the DNR form.” (P11: male, intensivist)*

Another participant mentioned the importance of apologizing before introducing sensitive topics that could be offensive to some patients.

*“We will say ‘I’m sorry, I don’t want to make it [death] come sooner, I just want to ask if your condition… you know, sometimes a person’s condition can improve, but sometimes it can deteriorate... I want to ask if… once more, I apologize for asking this, but what if your condition deteriorates, what will be your wishes?’.” (N15: female, palliative care nurse)*

#### Theme 3: Patients’ religious beliefs about the control and sanctity of life

Participants reported that patients’ religious beliefs play a significant role in their engagement in advance care planning. Patients who believed in God’s control over life may consider the concept of future planning to be contradictory with their beliefs.

*“We often communicate about the fact of their terminal condition and what could be their plans [for the end-of-life phase], but they [patients and their families] would argue that we would then be acting before God acts.” (N08: male, oncology nurse)*

The acknowledgement of patients’ religious beliefs and the incorporation of these beliefs into the conversation was reported to facilitate the rapport building necessary for advance care planning.

*“When initiating the conversation, we have to acknowledge their beliefs. After that, we need to acknowledge that we [doctors] are also humans. We have our limitations. We are not the solutions for every illness. If we do that, they are usually more open [to advance care planning] and accepting.” (P07: female, hepato-oncologist)*

Patients who believed that life is a sacred loan that should be protected were reported to often avoid conversations about limiting aggressive interventions. One participant shared her experience of using appropriate religious term to navigate the conversation, contextualize the message, and help clarify misconceptions. For instance, the use of the term *“mudarat,”* which means harm and is forbidden in an Islamic context, was reported to help Muslim patients better understand the concept of futile intervention and distinguish it from “giving up.”

*“We tell them that if we do this [futile] intervention, the ‘mudarat’ [harm] will be greater [than the benefit]. Doing harm to yourself is prohibited by our religion. This helps them appreciate our intention.*” *(N08: female, emergency care nurse)*

Nevertheless, while some participants reported their practice of integrating religious beliefs in the conversations, another participant felt the need to involve spiritual care providers to facilitate such a conversation.

#### Theme 4: The availability of a support system for advance care planning

Most participants reported their need for clear recommendations and guidelines for advance care planning, particularly pertaining to who should take the role in delivering it.

*“We frequently have these patients [appropriate for advance care planning], but as long as the primary physicians don’t feel the need to consult [a palliative care team], then this conversation will not occur.” (N10: female, oncology nurse)*

*“The way I see it, most of the time, we don’t know to which caregiver the patient should be referred [for advance care planning]? A psychiatrist? A spiritual caregiver? Hospice or palliative care team?.” (P05: male, pulmonologist)*

They also reported the need for a formal law to safeguard them from the legal consequences of engaging in advance care planning. Additionally, participants mentioned that integrating advance care planning into financial platforms would be essential to ensure patients’ access to it.

*“Unfortunately, in this hospital, it [advance care planning] is not covered by the national health insurance yet.” (N02: male, oncology nurse)*

The workload and time constraints were mentioned as important barriers to advance care planning. Also, the paper-based medical record system in a majority of Indonesian healthcare facilities hampered the accessibility and accountability of advance care planning related documents.

*“One day, I had documented the conversation, but when we wanted to retrieve the document upon the patient’s admission, it was gone.” (P10: female, geriatrician)*

Participants reported that patients’ opportunities for timely engagement in advance care planning was reduced by their late presentation to medical facilities – a common problem. Therefore, awareness of the benefits of advance care planning should be raised in the community.

*“Educating communities about advance care planning is important so they know that they have the right [to decide for themselves]” (P01: female, palliative care physician)*

Lastly, participants argued that patients’ health literacy would influence their ability to understand and appreciate the aim of advance care planning.

## Discussion

Our study showed that several Indonesian healthcare professionals were open to advance care planning but also considered that cultural sensitivity mattered to their engagement in it. They believed that its uptake would be facilitated by the family’s support for advance care planning, and for culturally sensitive communication, contextualization of advance care planning within the patient’s religious beliefs, and the establishment of public education, financial support, a legal platform, and proper training.

Being strongly collectivists, Indonesian people consider the maintenance of social harmony crucial [20–23]. Our study showed that, due to families’ leading role and their hierarchical structure, which may complicate advance care planning, healthcare professionals considered that gaining families’ support in advance care planning was essential to ensuring patients’ engagement in it. Therefore, the initial step towards patients’ successful engagement in advance care planning included careful consideration of family dynamics and how these may facilitate the conversation without disrupting the harmonious relationship between doctor, patient, and families. Indonesian culture is characterized by its indirect communication style, which prioritizes the maintenance of other people’s honour [24]. Our study showed that the use of indirect and positive terms was preferred both by healthcare professionals and by their patients. Available evidence has shown that patients’ preferences for communication approach vary across different cultures. For instance, Asian immigrants in Western countries [25, 26] and Japanese patients [27] were likely to prefer implicit communication. In contrast, Western patients preferred information delivered straight to the point and professionally [28]. They appreciated open discussion about how much detailed information they would want [29]. Our study also showed that offering an apology before introducing sensitive issues was another strategy that was reported to initiate advance care planning. Great caution should be exercised when approaching this conversation indirectly, as the main aim – exploring patients’ values – must still be attained [1]. Therefore, it is essential to develop a special training on end-of-life-related conversation into current medical curricula for healthcare professionals in Indonesia.

Our study showed that religious belief was considered as an important factor due to its role in facilitating message interpretation and its meaning-making among religiously devoted patients. Exploration and use of these patients’ beliefs in navigating the advance care planning conversation were believed to facilitate its uptake. Furthermore, our study suggested that Indonesian healthcare professionals believed that patients appreciated conversations about their religious beliefs. This finding supports the emerging evidence that religiosity does not necessarily negate the desire for prognostic communication and preparation for the end of life [30, 31]. It also supports the importance of the spiritual dimension of palliative care in Indonesia [32–34].

Lastly, our findings showed an urgent need for advance-care capacity building planning in Indonesia, which lagged behind other Asian countries [7]. In our study, the lack of agreement on the role of different healthcare professionals (nurses versus physicians, primary physicians versus palliative care team) in advance care planning led to a lack of leadership in it. Additionally, the lack of financial support inevitably hampered patients’ access and providers’ engagement. To aid the advocacy efforts on advance care planning, evidence of its value in Indonesia is needed.

### Implications

Our study indicates the importance of developing cultural sensitivity of advance planning. This requires healthcare professionals to create a meaningful understanding of the common features of patients’ cultures while avoiding stereotypical characterizations. An example of such a step is evaluating patients’ family dynamics and their communication norms, particularly when engaging with patients from a culture where family-centred decision-making and indirect communication are the norms. Likewise, in order to facilitate the engagement of religiously devoted patients, it is necessary to consider and contextualize their beliefs carefully.

### Strength and limitations

This study has several strengths. First, to capture a comprehensive range of participants’ experiences, we purposively sampled two types of healthcare professional. Second, the consistency of data collection was sustained by a single interviewer (DM) and observer (RD). However, several limitations must be considered when interpreting the findings. Firstly, it is possible that the use of a single interviewer led to systematic bias. However, this concern was addressed by ‘investigator triangulation’, which involved all researchers in analysing and discussing the findings. Secondly, we are aware that the participants’ meaning may have been clouded throughout the analysis by Indonesian-English language differences. Lastly, the study was undertaken in two tertiary, national referral hospitals, which limits its generalizability to other settings.

## Conclusion

Future directions for advance care planning in Indonesia should include sensitive cultural adaptation to the values of family harmony, communication norms, and religious beliefs. To complement current evidence and facilitate advocacy efforts, further study is needed on patients’ perspectives and the value of advance care planning in Indonesia.

## Supplementary Information

**Additional file 1: Appendix 1. **Guide for Focus-GroupDiscussions with Healthcare Professionals

## Data Availability

The original datasets generated and analyzed during the present study are not publicly available due to the requirement to preserve confidentiality. Upon reasonable request, the data material, which is in Indonesian language, is available in anonymized format from the corresponding author.
