# Supplementary material for: Opportunities and challenges for advance care planning in strongly religious family-centric societies: a Focus group study of Indonesian cancer-care professionals
Source: BMC Palliat Care. 2022 Jun 22;21:110. doi: 10.1186/s12904-022-01002-6 (PMC9215088; doi:10.1186/s12904-022-01002-6)
Supplement: Supplementary file 1 — Additional file 1: Appendix 1. Guide for Focus-GroupDiscussions with Healthcare Professionals [file 12904_2022_1002_MOESM1_ESM.docx]

**Appendix 1**

**Guide for Focus-Group Discussions with Healthcare Professionals**

1. The researcher introduces her/himself to the respondents and explains the study using the written (informed consent) form.
2. The researcher asks the respondents if they are willing to participate in the study.
3. The researcher asks the respondents to agree to audiotaping of the discussion process.
4. The researcher asks whether participants have heard about and understood the concept of advance care planning.
5. The researcher explains the concept and definition of advance care planning established in the Consensus of the European Association for Palliative Care.
6. The researcher elicits the respondents’ perspectives on advance care planning.
7. The researcher asks whether and how the respondents engage in advance care planning.
8. The researcher asks about the barriers and challenges of offering and practicing advance care planning in their current practice.
9. The researcher encourages discussion within the group.
10. At the end of the discussion, the researcher provides a summary of it.
